# Retraction: Knockdown of Sox2 inhibits OS cells invasion and migration via modulating Wnt/β-catenin signaling pathway

**DOI:** 10.3389/pore.2026.1612565

**Published:** 2026-08-13

**Authors:** Pathology and Oncology Research Editorial Office

Following publication, the authors contacted the Editorial Office to request the retraction of the cited article, stating that improper cropping of experimental images in Figure 3A (control group). The findings/conclusions reported in the article are no longer supported by the analyses/data. An investigation was conducted in accordance with POR policies that confirmed this; therefore, the article has been retracted.

This retraction was approved by the Editor-in-Chief of Pathology & Oncology Research. The authors agree to this retraction.

